# A Systematic Review and Meta-Analysis of Medial Meniscus Root Tears: Is Surgery the Key to Better Outcomes?

**DOI:** 10.7759/cureus.75199

**Published:** 2024-12-06

**Authors:** Ahmed Elnewishy, Abdelfatah M Elsenosy, Sam Nahas, Mohammad Abdalla, Naoum Symeon, Hagar Teama

**Affiliations:** 1 Trauma and Orthopaedics, Royal Berkshire Hospital, Reading, GBR; 2 Trauma and Orthopaedics, University Hospitals Dorset, Poole, GBR; 3 Trauma and Orthopaedics, Aneurin Bevan University Health Board, Newport, GBR; 4 Orthopaedics and Trauma Surgery, 251 Hellenic Air Force General Hospital, Athens, GRC; 5 Pharmacy, Kafr El Sheikh General Hospital, Kafr El Sheikh, EGY

**Keywords:** conservative management, ikdc, knee injury and osteoarthritis outcome score (koos), lysholm score, medial meniscus posterior root tear, meniscal repair, osteoarthritis progression, surgical repair, transtibial pull-out repair

## Abstract

Medial meniscus root tears (MMRTs) are serious injuries that disrupt knee biomechanics, often accelerating cartilage degeneration and osteoarthritis when left untreated. These injuries are increasingly recognized as a major cause of knee pain and functional limitations, particularly among middle-aged and older adults. This systematic review and meta-analysis aimed to evaluate the outcomes of conservative management compared to surgical intervention for MMRT, focusing on pain relief, functional recovery, and the progression of osteoarthritis. A thorough search of PubMed, Scopus, Google Scholar, and the Cochrane Library identified six studies that directly compared surgical repair, primarily transtibial pull-out repair, with conservative management. Outcome measures included the Knee Injury and Osteoarthritis Outcome Score (KOOS), the Lysholm score, and the International Knee Documentation Committee (IKDC) subjective score. Surgical intervention showed marked superiority in KOOS scores, with a standardized mean difference (SMD) of 1.42 (95% CI: 0.97 to 1.88, P < 0.00001), reflecting significant improvements in pain, daily function, and quality of life. However, pooled analyses for the Lysholm score (SMD: 0.21, 95% CI: -0.23 to 0.65, P = 0.35) and IKDC score (SMD: 0.12, 95% CI: -0.56 to 0.80, P = 0.73) did not show statistically significant differences between treatments. High heterogeneity (I² > 50%) was noted, likely due to differences in study populations, follow-up periods, and methodologies. These results suggest that surgical repair offers superior pain relief and functional benefits for MMRT compared to conservative management, positioning it as the preferred option for most patients. Nonetheless, conservative management may remain suitable for certain patients, particularly those with contraindications to surgery. Further high-quality, long-term research is essential to confirm these findings and inform clinical decision-making.

## Introduction and background

Medial meniscus root tears (MMRTs) represent a significant challenge in orthopedic practice due to their detrimental effects on knee biomechanics and overall joint health. These injuries are closely linked to the rapid onset of osteoarthritis (OA), as they compromise the meniscus's essential role in converting axial loads into hoop stresses, which is key to absorbing impact and maintaining knee stability [1]. MMRTs are known to increase peak contact pressures within the medial compartment, leading to cartilage damage and meniscal extrusion, both of which are major contributors to degenerative joint changes over time [2]. Emerging evidence underscores the importance of timely diagnosis and intervention, as untreated MMRTs can mimic the severe consequences of a total meniscectomy, significantly accelerating the progression toward total knee arthroplasty (TKA) [3].

MMRTs are biomechanically equivalent to losing the entire meniscus, making the knee joint highly vulnerable to increased wear and tear. This highlights the importance of timely repair when the articular cartilage remains intact [4]. Repair strategies, particularly the transtibial pull-out technique, have shown favorable outcomes in restoring knee stability and preventing OA progression, positioning MMRT repair as a cost-effective and clinically superior alternative to meniscectomy or conservative management [5].

Recent studies have identified various risk factors for MMRTs, emphasizing the significance of anatomical and biomechanical characteristics. A steep posterior tibial slope and shallow medial tibial plateau depth are associated with a higher incidence of MMRTs, as these features increase stress on the medial meniscus during knee movement [6]. Additionally, obesity is a well-established risk factor, with evidence linking higher body mass indices (BMI) to accelerated OA progression and an increased likelihood of spontaneous osteonecrosis following MMRT, particularly in patients with high Fracture Risk Assessment Tool (FRAX) scores, which assess osteoporotic fracture risk [7].

MMRTs are strongly correlated with meniscal extrusion, which exacerbates knee joint loading and cartilage damage, accelerating OA progression. Larger tear gaps significantly increase meniscal extrusion, further compounding cartilage wear and contributing to OA development [8]. Individuals engaging in high-impact or contact sports are at heightened risk for MMRTs, particularly when concurrent ligament injuries, such as anterior cruciate ligament (ACL) tears, are present. In such cases, both ACL and medial meniscus ramp lesions are frequently observed, which further destabilizes the knee [9].

Conservative management is often the first-line approach for meniscal tears, particularly in older patients or those with degenerative changes, as it avoids the increased risk of OA linked to surgical removal of meniscal tissue. Physical therapy is a cornerstone of conservative management, demonstrating efficacy in improving function and reducing pain for many patients with degenerative meniscal tears [10]. In cases where physical therapy and other conservative measures fail, some studies have explored regenerative treatments, such as platelet-rich plasma (PRP) and micro-fragmented adipose tissue injections. These therapies have shown promise in reducing pain and improving knee function for up to a year, though further rigorous studies are needed to confirm their long-term efficacy [11].

Patient perceptions regarding treatment also influence outcomes. Many patients with degenerative meniscal tears view surgery as a more definitive solution than conservative care. This belief, often shaped by MRI findings and expectations from surgical consultations, highlights the need to educate patients on the efficacy and safety of conservative management, particularly in cases where surgery is unlikely to offer additional benefits [12].

Emerging evidence suggests that conservative treatments, while effective initially, may sometimes require supplementation or replacement with surgical intervention, particularly in patients with advanced degenerative changes or complex tear patterns. For instance, the European Society of Sports Traumatology, Knee Surgery, and Arthroscopy (ESSKA) consensus advises that preserving meniscal tissue should remain the first-line treatment to delay joint degeneration and OA onset [13].

Surgical intervention, particularly through root repair techniques, has shown promising results in reducing the risk of OA and preserving knee function. Meniscus root repair is often favored over meniscectomy or nonoperative approaches due to its lower rates of OA and TKA. Techniques like the transtibial pull-out repair effectively restore the meniscus's load-distributing function, which is crucial for maintaining knee health [5].

For patients with varus alignment, combining medial meniscus root repair with high tibial osteotomy (HTO) has demonstrated better long-term outcomes and improved load distribution across the knee. Studies comparing isolated root repair, HTO, and combined approaches suggest that HTO, either alone or combined with root repair, offers lasting improvements in knee function, particularly in patients with varus deformities [14]. Additionally, biological augmentations, such as synovial mesenchymal stem cell (MSC) transplantation, have emerged as promising adjuncts for enhancing healing in complex meniscus repairs. Early case studies indicate that MSCs improve postoperative outcomes and pain management, especially in degenerative cases where traditional repair methods may fail [15].

Comparative studies strongly favor meniscus root repair over conservative and partial meniscectomy approaches for MMRTs. Research consistently shows that root repair significantly reduces OA progression rates and subsequent TKA compared to conservative treatment and meniscectomy, especially in cases where restoring hoop tension is critical for joint stability [16]. Moreover, a 10-year follow-up study revealed better clinical scores and lower TKA rates for meniscus repair versus meniscectomy, solidifying repair as the preferred long-term solution for joint preservation [17].

While conservative treatments may offer initial symptom relief for middle-aged patients, they often fail to prevent OA progression, particularly in cases with degenerative MMRTs. A review found no significant long-term advantage of meniscectomy over conservative management, noting that meniscectomy can accelerate OA progression due to the loss of meniscal function [18]. By contrast, repair options like the transtibial pull-out technique effectively restore joint stability and reduce OA risk, making them a cost-effective choice for patients who might otherwise face greater healthcare costs from conservative or meniscectomy approaches [5].

Determining the optimal treatment for MMRTs remains challenging, particularly regarding combined approaches and the influence of individual factors such as alignment and cartilage health. Further research is needed to tailor treatment strategies and delay arthritis progression.

## Review

Objective

The objective of this review was to evaluate and compare the effectiveness of conservative management and surgical interventions for MMRTs.

Methods

Search Strategy

A structured search was conducted in November 2024 across PubMed, Scopus, Google Scholar, and the Cochrane Library to identify studies comparing surgical and conservative treatments for MMRTs. The search incorporated combinations of MeSH terms and keywords such as “medial meniscus root tear”, “conservative treatment”, “transtibial pull-out repair”, and “knee function”. Boolean operators (AND, OR) were utilized to refine results, and filters restricted the search to English-language studies published within the last decade. Additionally, reference lists of selected articles were manually reviewed to identify any additional relevant studies.

Inclusion Criteria

Eligible studies included randomized controlled trials (RCTs), cohort studies, case series, and observational studies that directly compared surgical repair, particularly the transtibial pull-out technique, with conservative management approaches (e.g., physical therapy and nonsteroidal anti-inflammatory drugs (NSAIDs)). Included studies were required to report on primary outcomes such as the Knee Injury and Osteoarthritis Outcome Score (KOOS), Lysholm score, or International Knee Documentation Committee (IKDC) subjective score. Only studies published in English were considered.

Exclusion Criteria

Studies that did not directly compare surgical and conservative interventions, along with case reports, editorials, and opinion articles, were excluded. Further exclusions were applied to studies lacking sufficient outcome data or those not available in English.

Outcome Measures

Primary outcomes included KOOS, Lysholm, and IKDC scores, which assess pain relief, knee function, and patient-reported outcomes. Secondary outcomes included the progression of OA, conversion rates to TKA, and patient satisfaction. These measures provided a comprehensive evaluation of both the functional and structural effectiveness of the treatments.

Data Extraction and Quality Assessment

Two independent reviewers systematically extracted data using a standardized form to capture key study characteristics, including design, patient demographics, interventions, outcomes, and follow-up durations. Any disagreements were resolved through consensus or consultation with a third reviewer. Study quality was assessed using the Grading of Recommendations, Assessment, Development, and Evaluation (GRADE) framework, evaluating the risk of bias, consistency, directness, and precision. Each study was categorized as high, moderate, low, or very low quality based on these domains.

Statistical Analysis

Statistical analysis was performed using RevMan version 5.4 software (Cochrane Collaboration, London, UK) [19]. For continuous variables such as KOOS, Lysholm, and IKDC scores, standardized mean differences (SMDs) with 95% confidence intervals (CIs) were calculated. A random-effects model was employed to address substantial heterogeneity, indicated by an I² statistic above 50%. Publication bias was assessed using funnel plots and Egger’s test, with statistical significance set at P < 0.05.

Results

Search and Study Selection

Our structured search identified 200 records. After removing duplicates, 170 records remained for the initial screening. During the title and abstract review, we excluded 130 studies that did not meet the specific inclusion criteria focused on comparing conservative and surgical interventions for MMRTs, resulting in 40 full-text articles for detailed review.

**Figure 1:**
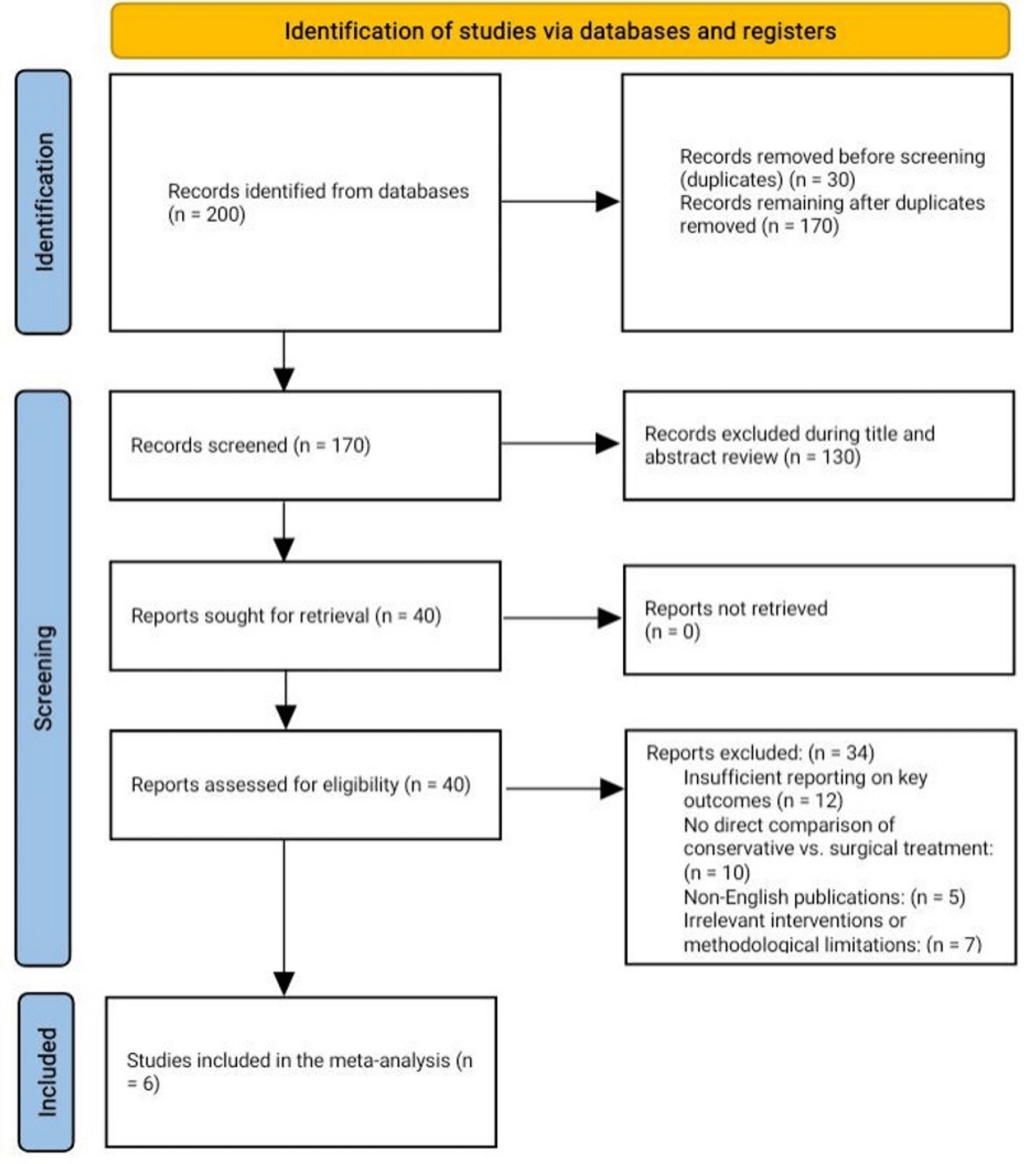
PRISMA flowchart of the reviewed studies. PRISMA: Preferred Reporting Items for Systematic Reviews and Meta-Analyses.

Following an in-depth assessment, 34 articles were excluded. Reasons for exclusion included studies that did not provide a direct comparison of conservative versus surgical treatment approaches, insufficient reporting on primary outcomes (e.g., KOOS pain and Lysholm scores), or non-English publications. Additional exclusions were applied to studies with irrelevant interventions or methodological limitations. Ultimately, six studies fulfilled all the inclusion criteria and were included in the final quantitative synthesis (meta-analysis) (Figure 1).

Study Characteristics

The six studies included in this meta-analysis varied in design, sample size, and patient demographics. The studies comprised RCTs, cohort studies, and retrospective comparative analyses, focusing on patients with MMRTs. Sample sizes ranged from 38 to 150 patients, with a total of 488 patients included across all studies (238 in the surgical group and 250 in the conservative treatment group).

The populations studied primarily consisted of middle-aged adults with MMRTs, often with varying degrees of OA but without advanced OA or severe knee malalignment. Interventions in the surgical group primarily included the transtibial pull-out repair technique, while the conservative group received nonoperative management consisting of physical therapy, activity modification, and NSAID use.

Follow-up periods varied among the studies, with durations spanning from 1.5 years to an average of six years. This allowed for a comprehensive evaluation of both short- and long-term outcomes. The primary outcomes measured included the KOOS pain subscale, the Lysholm score, and the IKDC subjective score, providing insight into pain levels and functional recovery. Secondary outcomes included the incidence of OA progression, the rate of TKA conversion, and patient-reported satisfaction (Table 1).

**Table 1:** Data extraction table for the reviewed studies. MMRT: medial meniscus root tear; ACL: anterior cruciate ligament; KL: Kellgren-Lawrence; NSAIDs: nonsteroidal anti-inflammatory drugs; KOOS: Knee Injury and Osteoarthritis Outcome Score; ADL: activities of daily living; Sport/Rec: sports and recreation; QoL: quality of life; EQ-5D: EuroQol 5 Dimension; EQ-VAS: EuroQOL Visual Analog Scale; IKDC: International Knee Documentation Committee; ICRS: International Cartilage Repair Society; SF-36: 36-Item Short Form Survey; GPE: Global Perceived Effect; VAS: visual analog scale; PCS: Physical Component Summary; MCS: Mental Component Summary; OA: osteoarthritis; MA: mechanical axis.

Category	Gauffin et al. [20]	Dragoo et al. [21]	Ahn et al. [22]	Pan et al. [23]	Roos et al. [24]	Lee et al. [18]
Study design	Prospective, randomized controlled trial (RCT)	A cohort study comparing arthroscopic all-inside meniscus root repair (AR) vs. nonoperative observation (O)	Retrospective comparative study comparing conservative treatment vs. arthroscopic pull-out repair for MMRT	Retrospective comparative study comparing repair vs. untreated posterior lateral meniscus root (PLMR) tears in ACL reconstruction	A sham-controlled randomized trial comparing arthroscopic partial meniscectomy (APM) vs. sham (skin incisions only)	Retrospective comparative study comparing conservative treatment vs. meniscectomy for degenerative medial meniscus posterior root tears (MMPRT)
Sample size	150 patients, aged 45-64 years	48 patients (30 in the AR group, 18 in the O group)	38 patients (25 in the pull-out repair group, 13 in the conservative treatment group)	62 patients (31 with PLMR repair, 31 untreated)	44 patients (22 in each group) aged 35-55 years	146 patients (90 in the meniscectomy group, 56 in the conservative group)
Level of evidence	Level 1	Level 2	Level 3	Level 3	Level 1	Level 3
Patient demographics	Middle-aged patients with meniscal symptoms, aged 45-64 years, without severe osteoarthritis	Patients >45 years with moderate osteoarthritis (KL grade 2) and meniscus root tears	Patients with medial meniscus root tear (MMRT); mean age 55.6 years (repair) vs. 62.3 years (conservative)	Patients with ACL and PLMR tears; mean age 28 years; matched on age, BMI, sex, and follow-up time	Patients aged 35-55 with MRI-verified medial meniscus tear, no severe osteoarthritis	Middle-aged patients with degenerative MMPRT, without advanced osteoarthritis or significant malalignment
Intervention details	3-month exercise program (nonsurgery group) vs. exercise + knee arthroscopic surgery (surgery group)	AR group: All-inside suture technique with an additional mattress suture for stabilization. O group: Nonoperative management with activity modification, physical therapy, and NSAIDs	Pull-out repair group: Double transosseous pull-out suture technique. Conservative group: Pain control, physical therapy, strengthening exercise	Repair group (Group A): Transtibial pull-out suture technique for PLMR repair. Control group (Group B): No PLMR repair, only ACL reconstruction	APM group: Arthroscopic partial meniscectomy. Sham group: Skin incisions only (no internal intervention)	Meniscectomy group: Arthroscopic partial meniscectomy. Conservative group: NSAIDs, physical therapy, and activity modification
Follow-up duration	3 years	Minimum 2 years (average follow-up of 4.4 years for AR group and 4.0 years for O group)	Average 17.4 months (repair group) and 18.4 months (conservative group)	Minimum 2 years	2 years (with assessments at 3 and 24 months)	Average 6.3 years
Outcome measures	Primary outcome measure: KOOS pain subscore. Secondary outcome measures: KOOS subscales (symptoms, ADL, Sport/Rec, QoL), EQ-5D Index, EQ-VAS	KOOS subscales, Lysholm score, Tegner activity score, Veterans RAND 12-Item Health Survey (VR-12)	IKDC subjective score, Tegner and Lysholm activity scale	Lysholm score, subjective IKDC score, ICRS classification on radiographic grading of osteoarthritis	KOOS (KOOS5 mean score and subscales: pain, symptoms, ADL, Sports/Rec, QoL), EQ-5D, SF-36, GPE score, one-leg hop, knee bend test, isometric knee extensor strength	VAS (pain), IKDC subjective score, Tegner activity scale, Lysholm knee score, KL grade for osteoarthritis progression
Results	KOOS pain subscore, surgery: Significant improvement at 3 years (29.1 points; 95% CI: 23.6–34.5; P < 0.001). Nonsurgery: Significant improvement (21.4 points; 95% CI: 15.1–27.7; P < 0.001). Between-group difference: Not significant at 3 years (7.6 points; 95% CI: –0.6 to 15.9; P = 0.068). KOOS symptoms subscore, surgery: Improved at 3 years (25.2 points; 95% CI: 20.1–30.2). Nonsurgery: Improved (16.8 points; 95% CI: 11.2–22.5). Significant difference favoring surgery (8.3 points; P = 0.029). KOOS ADL subscore, surgery: Improved at 3 years (22.1 points; 95% CI: 17.6–26.6). Nonsurgery: Improved (15.6 points; 95% CI: 9.7–21.5). Between-group difference: Not significant (6.5 points; P = 0.080). KOOS Sport/Rec subscore, surgery: Improved (33.7 points; 95% CI: 26.4–41.0). Nonsurgery: Improved (29.3 points; 95% CI: 21.4–37.1). Between-group difference: Not significant (4.4 points; P = 0.406). KOOS QoL subscore, surgery: Improved (38.4 points; 95% CI: 31.6–45.2). Nonsurgery: Improved (29.4 points; 95% CI: 22.8–36.0). Between-group difference: Not significant (9.0 points; P = 0.062). EQ-5D Index, surgery: Improved to 0.88 (95% CI: 0.84–0.92). Nonsurgery: Improved to 0.78 (95% CI: 0.71–0.85). Significant improvement favoring surgery (0.13 points; P = 0.016) EQ-VAS, surgery: Improved to 79 (95% CI: 74–84). Nonsurgery: Improved to 76 (95% CI: 71–81). Between-group difference: Not significant (3.4 points; P = 0.353). Subgroup analysis - Age: Patients ≥55 years showed greater improvement in KOOS pain scores than younger patients, regardless of treatment - Mechanical symptoms: patients without mechanical symptoms improved more after surgery (KOOS pain: 18.6 points; P = 0.008), while patients with mechanical symptoms did not show a significant difference (P = 0.652). Adverse events: Minimal adverse effects; a few patients in the surgery group required additional procedures, with no major complications reported.	KOOS pain, AR: Improved by 32.0 points (P < 0.001). O: Improved by 15.7 points (P = 0.003). Between-group: Significant improvement in the AR group (P = 0.009). KOOS Symptoms, AR: Improved by 24.2 points (P < 0.001). O: Improved by 9.3 points (P = 0.070). Between-group: Significant improvement in the AR group (P = 0.029). KOOS ADL, AR: Improved by 27.3 points (P < 0.001). O: Improved by 15.4 points (P = 0.006) Between-group: Not significant (P = 0.064). KOOS Sport/Rec, AR: Improved by 28.8 points (P < 0.001). O: Improved by 20.6 points (P = 0.157). Between-group: Not significant (P = 0.466). KOOS QoL, AR: Improved by 27.3 points (P < 0.001). O: Improved by 13.9 points (P = 0.075). Between-group: Not significant (P = 0.096). Lysholm score, AR: Improved by 27.3 points (P < 0.001). O: Improved by 7.1 points (P = 0.248). Between-group: Significant improvement in the AR group (P = 0.016). VR-12 PCS, AR: Improved by 9.9 points (P < 0.001). O: Improved by 6.0 points (P = 0.038). Between-group: Not significant (P = 0.259). VR-12 MCS, AR: No significant change (-0.2, P = 0.855). O: No significant change (-1.5, P = 0.274). Between-group: Not significant (P = 0.302). Tegner score, AR: Improved by 0.7 points (P = 0.002). O: No significant change (0.0, P = 0.745). Between-group: Not significant (P = 0.104). TKA conversion, AR: 3.3% converted to TKA. O: 33.3% converted to TKA. Between-group: The AR group had a significantly lower conversion rate (P = 0.008); a 93.2% lower hazard ratio for TKA in the AR group (P = 0.013).	IKDC subjective score, Repair group: Improved from 37.3 to 59.2 (P < 0.001). Conservative group: Slight increase from 44.7 to 45.9 (P = 0.633). Between-group difference: Significant improvement in the repair group (P < 0.001). Tegner and Lysholm scale, Repair group: Improved from 57.3 to 73.4 (P < 0.001). Conservative group: Decreased slightly from 51.6 to 51.2 (P = 0.932). Between-group difference: Significant improvement in the repair group (P = 0.017). Prognostic factors, varus alignment (MA angle > 5°): severe varus alignment associated with poorer outcomes. Cartilage degeneration (Outerbridge Grade III/IV): severe degeneration linked to worse results in the repair group (P < 0.001). Subgroup analysis, patients with mild varus (MA ≤ 5°) and mild cartilage degeneration (Grades I/II) showed better outcomes compared to severe varus and severe cartilage degeneration. Adverse events: none reported.	Lysholm score, Group A: Improved from 59.03 ± 19.17 to 92.34 ± 6.32 (P < 0.001). Group B: Improved from 58.35 ± 18.14 to 87.77 ± 11.99 (P < 0.001). Between-group difference: Not significant postoperatively (P > 0.05). IKDC subjective score, Group A: Improved from 62.08 ± 19.21 to 90.06 ± 8.59 (P < 0.001). Group B: Improved from 63.27 ± 18.73 to 86.86 ± 11.47 (P < 0.001). Between-group difference: Not significant postoperatively (P > 0.05). Radiographic evaluation, Group A: 80% had normal/mild osteoarthritis (ICRS grades 0–1). Group B: 48% had normal/mild osteoarthritis (ICRS grades 0–1). Between-group difference: Significant difference favoring repair (P = 0.003). Functional outcomes: Group A showed a trend toward better functional outcomes, though not statistically significant for Lysholm and IKDC scores postoperatively. Adverse events: No significant complications; no postoperative infections or joint stiffness reported.	KOOS5, APM: 21.8-point improvement (95% CI: 11.2–32.4). Sham: 13.6-point improvement (95% CI: 3.0–24.2). Between-group difference: 8.2 points, not statistically significant (95% CI: –3.4 to 19.8; P = 0.161). KOOS pain, APM: 23.9-point improvement (95% CI: 12.9–34.9). Sham: 14.0-point improvement (95% CI: 2.7–25.2). Between-group difference: 9.9 points (P = 0.110). KOOS symptoms, APM: 16.8-point improvement (95% CI: 6.6–27.0). Sham: 10.2-point improvement (95% CI: –0.0–20.5). Between-group difference: 6.6 points (P = 0.240). KOOS ADL, APM: 20.0-point improvement (95% CI: 10.4–29.6). Sham: 11.0-point improvement (95% CI: 1.3–20.7). Between-group difference: 9.0 points (P = 0.095). KOOS Sport/Rec, APM: 18.5-point improvement (95% CI: 2.5–34.5). Sham: 12.9-point improvement (95% CI: –3.9–29.7). Between-group difference: 5.6 points (P = 0.529). KOOS QoL, APM: 23.1-point improvement (95% CI: 12.0–34.1). Sham: 11.5-point improvement (95% CI: 0.3–22.7). Between-group difference: 11.5 points (P = 0.062). EQ-5D VAS, APM: 5.8-point improvement. Sham: 12.4-point improvement. Between-group difference: –6.6 points (P = 0.164). SF-36 Physical Component, APM: 7.9 points. Sham: 5.8 points. Between-group difference: 2.1 points (P = 0.452). Global Perceived Effect, Better/Much Better: APM = 67%, Sham = 37%. Between-group difference: P = 0.059. Functional measures: No significant differences in the one-leg hop test, knee bend test, or isometric knee extensor strength between groups. Adverse events: 11 adverse events in total: 6 in the APM group (including 2 re-arthroscopies), 3 in the Sham group, with 2 crossovers to APM.	VAS pain score, Meniscectomy group: Reduced from 5.9 to 4.3 (P < 0.001). Conservative group: Reduced from 4.3 to 3.8 (P = 0.01). Between-group difference: Not significant at final follow-up (P = 0.07). IKDC subjective score, Meniscectomy group: Improved from 26.3 to 33.9 (P < 0.001). Conservative group: Improved from 30.6 to 38.1 (P = 0.01). Between-group difference: Not significant (P = 0.18). Tegner activity scale, Meniscectomy group: Improved from 2.3 to 2.8 (P < 0.001). Conservative group: Improved from 2.7 to 3.1 (P = 0.03). Between-group difference: Not significant (P = 0.08). Lysholm score, Meniscectomy group: Improved from 50.9 to 65.5 (P < 0.001). Conservative group: Improved from 54.1 to 67.0 (P < 0.001). Between-group difference: Not significant (P = 0.53). Radiographic outcomes (OA progression): Greater osteoarthritis progression in the meniscectomy group (P = 0.03). Higher varus angle (P = 0.04) and narrower joint space in the meniscectomy group (P = 0.03). Survivorship analysis, Meniscectomy group: 10-year survival rate of 87%. Conservative group: 10-year survival rate of 88%. Between-group difference: No significant difference in conversion to knee arthroplasty (P = 0.8).
Conclusions	The initial benefit of knee arthroscopic surgery observed at 1 year diminished at the 3-year follow-up. Both groups (surgery and nonsurgery) showed sustained improvement in KOOS pain and other outcomes, suggesting that exercise alone may provide similar long-term improvements for middle-aged patients with meniscal symptoms.	The AR group demonstrated greater improvements in KOOS pain, symptoms, and Lysholm scores, with a lower rate of TKA conversion, suggesting AR is an effective treatment for meniscus root tears in older patients with moderate osteoarthritis.	Pull-out repair provided better clinical outcomes compared to conservative treatment in patients with MMRT, except in cases with severe varus alignment and severe cartilage degeneration where results were similar to conservative treatment.	Both surgical and conservative approaches improved knee function, but surgical repair of PLMR resulted in a trend toward higher functional scores and lower rates of osteoarthritis compared to untreated PLMR.	Arthroscopic partial meniscectomy showed greater improvement in KOOS5 and individual KOOS subscales over skin incisions only at 2 years. However, the study was underpowered, with many non-blinded participants and crossovers in the sham group. Results suggest a modest benefit of APM, but limitations reduce generalizability to a broader patient population.	Both conservative treatment and meniscectomy provided symptomatic relief for MMPRT, with no significant difference in functional outcomes at the final follow-up. However, osteoarthritis progression was more severe in the meniscectomy group, suggesting that meniscectomy may offer no advantage over conservative treatment for degenerative MMPRT in middle-aged patients.

Quality Assessment of Included Studies

The quality of the included studies was evaluated using the GRADE framework. This tool assesses the quality of evidence based on several key domains. Table 2 presents the GRADE-based quality assessment of the included studies.

**Table 2:** Quality assessment of studies using the GRADE framework. GRADE: Grading of Recommendations, Assessment, Development, and Evaluation; RCT: randomized controlled trial; CI: confidence interval.

Study	Risk of bias	Inconsistency	Indirectness	Imprecision	Publication bias	Overall quality
Gauffin et al. [20]	Moderate (RCT, with potential confounders)	Low (consistent results across similar RCTs)	Low (directly applicable)	Moderate (CI not tightly bound)	Low (well-documented RCT)	Moderate
Dragoo et al. [21]	High (cohort design; non-randomized)	Moderate (some variations across cohorts)	Low (directly related intervention)	High (small sample size, large CI)	Moderate (likely publication on positive outcomes)	Low
Ahn et al. [22]	High (retrospective, high confounding)	Moderate (consistency across some similar studies)	Low (relevant patient group)	Moderate (imprecision due to sample size)	Moderate (potential selective reporting)	Low to moderate
Pan et al. [23]	High (non-randomized, retrospective)	Low (findings are consistent with other repairs)	Low (directly relevant)	Moderate (limited sample size)	Moderate (publication trends)	Low
Roos et al. [24]	Moderate (randomized, some crossover)	Moderate (some outcome variance across interventions)	Low (focused on meniscus tear intervention)	Moderate (CI suggests need for larger study)	Low (controlled RCT)	Moderate
Lee et al. [18]	High (observational, risk of bias)	Moderate (variability in conservative vs. meniscectomy results)	Low (focused on relevant tear type)	High (sample size limits precision)	Moderate (possible reporting bias)	Low

Results of meta-analysis

KOOS Subscales

The total effect of surgical intervention across all KOOS subscales showed an SMD of 1.42 (95% CI: 0.97 to 1.88, P < 0.00001), indicating that surgery significantly outperformed conservative treatment in enhancing knee function and patient outcomes for MMRTs. Surgery demonstrated a marked improvement in pain relief, with an SMD of 1.68 (95% CI: 0.64 to 2.72, P = 0.002), suggesting a major reduction in pain levels in the surgical group. Symptom relief was similarly notable, with an SMD of 1.65 (95% CI: 0.15 to 3.15, P = 0.03), showing that surgery more effectively alleviated knee-related symptoms than conservative management.

**Figure 2:**
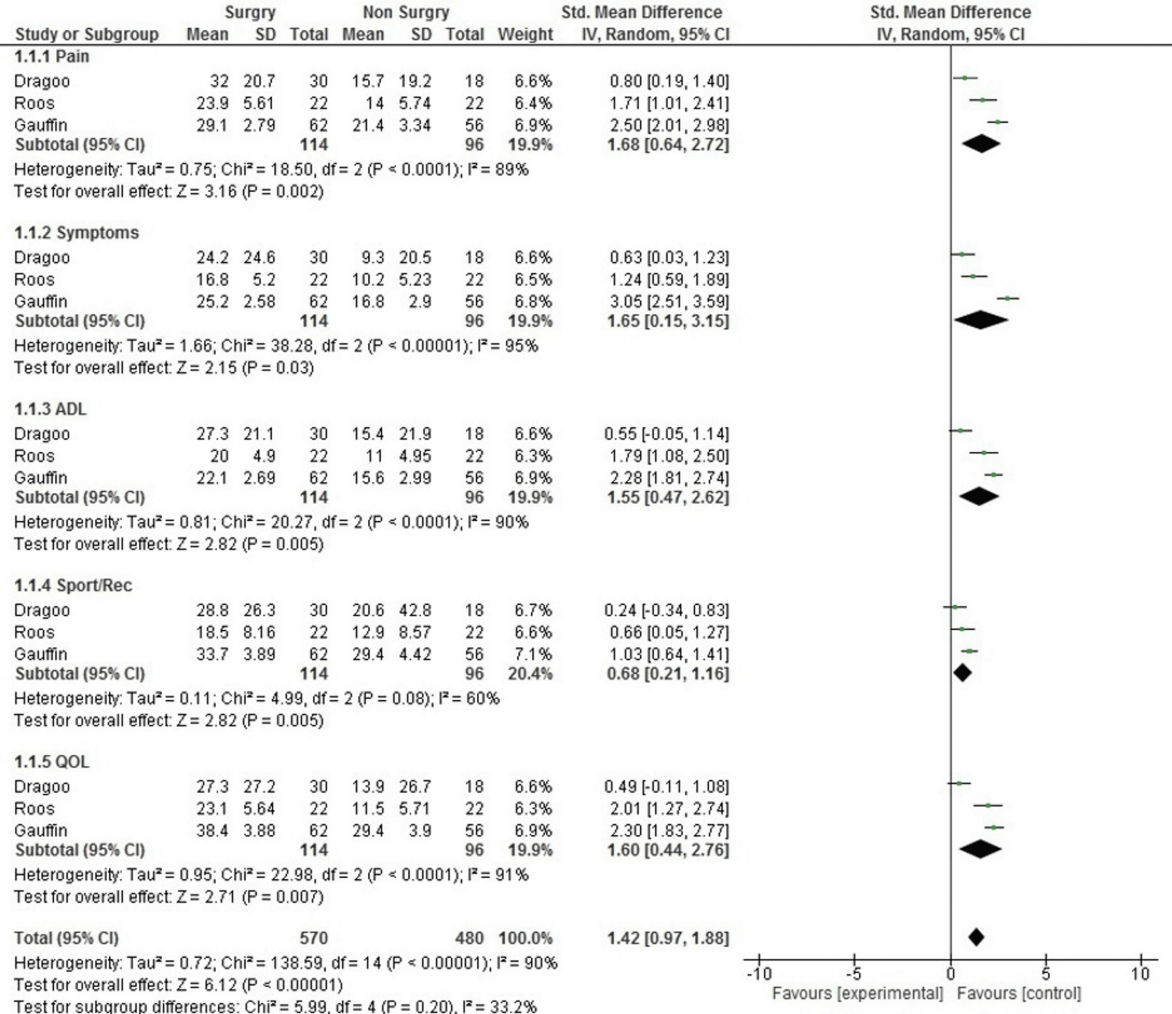
KOOS subscales forest plot. Comparison of KOOS subscales (pain, symptoms, ADL, Sport/Rec, and QoL) between surgical and conservative management of medial meniscus root tears. Data adapted from Dragoo et al. [21], Roos et al. [24], and Gauffin et al. [20]. The pooled analysis demonstrates the superiority of surgical intervention in key functional outcomes. KOOS: Knee Injury and Osteoarthritis Outcome Score; ADL: activities of daily living; Sport/Rec: sports and recreation; QoL: quality of life.

In terms of functional outcomes, the surgical group exhibited significant improvements in activities of daily living (ADL), with an SMD of 1.55 (95% CI: 0.47 to 2.62, P = 0.005), indicating better performance in routine tasks among patients who underwent surgery. Sports and recreational activities also saw moderate improvement, with an SMD of 0.68 (95% CI: 0.21 to 1.16, P = 0.005), suggesting that surgery provided some benefits in physical activity levels, though to a lesser extent than in other areas. Quality of life (QOL) was also significantly enhanced in the surgical group, with an SMD of 1.60 (95% CI: 0.44 to 2.76, P = 0.007), highlighting an overall improvement in well-being for surgical patients. The high heterogeneity across these outcomes (I² > 50%) justified the use of a random-effects model, which accounted for variations among studies (Figure 2).

Publication Bias for KOOS Subscales

**Figure 3:**
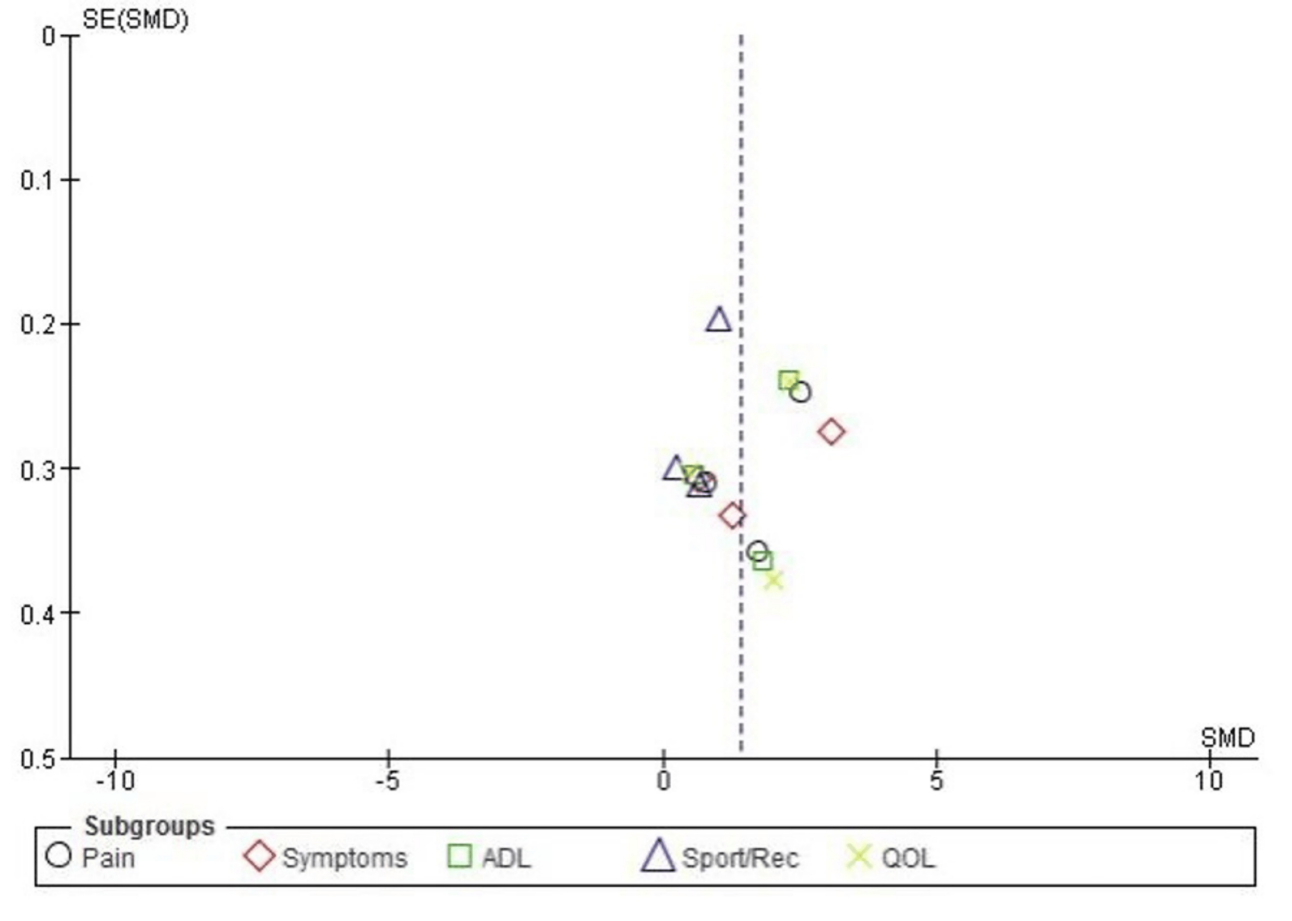
KOOS subscales funnel plot. KOOS: Knee Injury and Osteoarthritis Outcome Score; ADL: activities of daily living; Sport/Rec: sports and recreation; QoL: quality of life; SE: standard error; SMD: standardized mean difference.

The publication bias assessment for the KOOS subscales indicated minimal bias in this meta-analysis. The funnel plot appeared symmetrical, suggesting a balanced distribution of studies without significant missing data. Additionally, Egger’s test results (P > 0.05) further supported the lack of substantial publication bias, reinforcing the reliability of the findings across the subscales (Figure 3).

Lysholm Score

**Figure 4:**
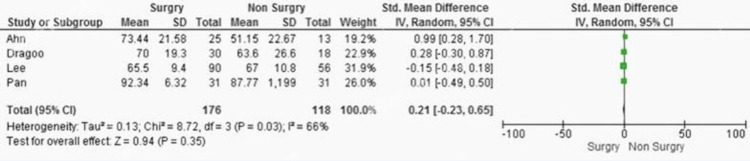
Lysholm score forest plot. Comparison of Lysholm scores between surgical and conservative management of medial meniscus root tears. Data adapted from Ahn et al. [22], Dragoo et al. [21], Lee et al. [18], and Pan et al. [23]. The forest plot illustrates the differences in functional outcomes, with square brackets indicating reference citations.

The meta-analysis for the Lysholm score comparing surgical and nonsurgical treatments across studies resulted in a pooled SMD of 0.21 (95% CI: -0.23 to 0.65, P = 0.35). This indicates no statistically significant difference between the two groups. Heterogeneity was moderate (I² = 66%), indicating variability across the studies, which could suggest differences in study populations, interventions, or methodologies (Figure 4).

Publication Bias for Lysholm Score

**Figure 5:**
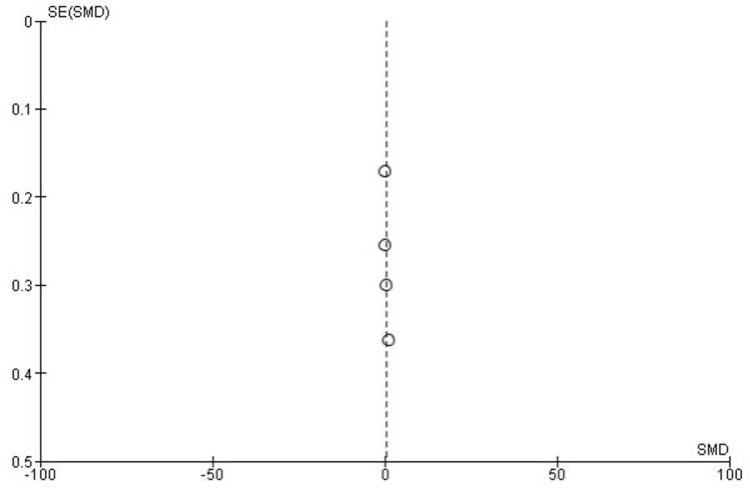
Lysholm score funnel plot. SE: standard error; SMD: standardized mean difference.

The publication bias for the Lysholm score meta-analysis was evaluated. The funnel plot displayed a symmetrical distribution of studies around the central line, indicating minimal publication bias. Egger’s test further supported this observation, with P > 0.05 (Figure 5).

IKDC Score

**Figure 6:**
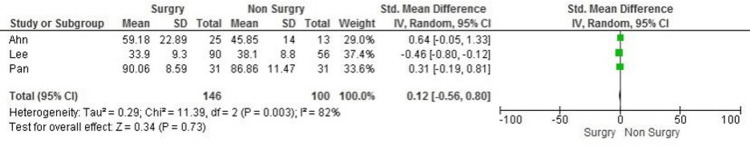
IKDC score forest plot. Comparison of IKDC subjective scores between surgical and conservative management of MMRT. Data adapted from Ahn et al. [22], Lee et al. [18], and Pan et al. [23]. The forest plot highlights the differences in knee function outcomes, with square brackets indicating reference citations. IKDC: International Knee Documentation Committee; MMRT: medial meniscus root tear.

The IKDC score, comparing surgical and nonsurgical treatments, produced a pooled SMD of 0.12 (95% CI: -0.56 to 0.80, P = 0.73). This result indicates no statistically significant difference between the surgical and conservative approaches. Heterogeneity was substantial (I² = 82%), indicating considerable variability among the included studies (Figure 6).

Publication Bias for IKDC Score

**Figure 7:**
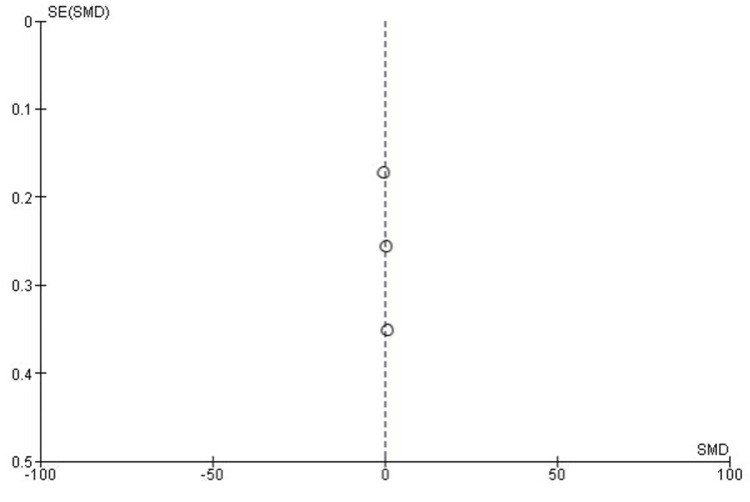
IKDC score funnel plot. IKDC: International Knee Documentation Committee; SE: standard error; SMD: standardized mean difference.

The funnel plot appeared symmetrical, with studies closely clustered around the center, indicating minimal likelihood of publication bias. Egger’s test further supported this observation, with P > 0.05 (Figure 7).

Discussion

This systematic review and meta-analysis provide a comprehensive comparison of surgical and conservative treatment strategies for MMRTs, with a focus on functional outcomes and the progression of OA. Recent studies consistently highlight that surgical repair, particularly techniques like the transtibial pull-out repair, yields superior long-term results compared to nonoperative management, especially in terms of preventing OA and preserving knee function.

The findings of this meta-analysis align with existing research emphasizing the benefits of surgical repair in improving joint stability and slowing the progression of OA, ultimately reducing the need for TKA. Faucett et al. [5] demonstrated that meniscus repair, as opposed to meniscectomy or conservative management, resulted in significantly lower rates of OA and TKA over a 10-year period, supporting its clinical and economic advantages. Similarly, Kwak et al. [25] reported that a larger meniscal extrusion ratio serves as a poor prognostic factor for conservative treatment, suggesting that patients with severe extrusion may derive greater benefits from early surgical intervention.

While surgical repair offers clear benefits, conservative management remains an appropriate option for specific patient populations, particularly those of advanced age or with early-stage OA. Kim et al. [26] identified factors such as age and meniscal extrusion as key determinants of OA progression in patients managed nonoperatively. Their findings suggest that conservative approaches may be suitable for select patients with minimal meniscal extrusion and advanced age. However, long-term data indicate that conservative treatment often leads to progressive joint degeneration, reinforcing surgical repair as the more effective option for younger, active individuals [21].

The choice of surgical technique also plays a crucial role in determining outcomes. The transtibial pull-out repair technique, commonly referenced in recent studies, demonstrates strong biomechanical stability by restoring hoop tension in the meniscus - a critical component for maintaining joint function. Krych et al. [4] highlighted that repair techniques effectively reduce OA progression and healthcare costs when compared to nonoperative approaches. However, fully halting OA progression remains challenging, as surgical repair alone may not completely restore normal joint mechanics.

These findings emphasize the importance of early diagnosis and timely intervention to safeguard joint health. Future research should prioritize long-term evaluations of different surgical methods, investigate the potential of biological augmentations such as stem cell therapy, and establish standardized treatment guidelines to aid patient selection. While conservative treatment may be adequate for older patients with lower physical demands, surgical repair is better suited for younger, active patients at higher risk of OA progression.

Limitations

This review’s findings are constrained by the high heterogeneity among the included studies, stemming from differences in sample sizes, study designs, and treatment protocols for conservative management, which complicates direct comparisons. The inclusion of both cohort studies and randomized trials may introduce bias, and the predominantly short- to medium-term follow-up durations limit insights into long-term outcomes. Future research should focus on standardizing treatment protocols and conducting longer-term studies to improve the reliability and applicability of future analyses.

## Conclusions

This review underscores the advantages of surgical intervention, particularly transtibial pull-out repair, for patients with medial meniscus root tears. Surgical repair consistently demonstrates better outcomes, including significant pain relief, improved functionality, and a reduced risk of osteoarthritis progression. Although conservative management may remain a reasonable option for specific groups, such as older patients or those with minimal meniscal extrusion, surgical repair should be the preferred choice for younger, more active individuals or those at a higher risk of OA progression. Tailored treatment decisions that account for individual factors, such as age, knee alignment, and the degree of meniscal extrusion, are essential to achieve optimal outcomes. Further research with extended follow-up periods and standardized treatment protocols is vital to confirm these findings and enhance clinical recommendations.
